# Occurrence of lactational mastitis and medical management: A prospective cohort study in Glasgow

**DOI:** 10.1186/1746-4358-3-21

**Published:** 2008-08-25

**Authors:** Jane A Scott, Michele Robertson, Julie Fitzpatrick, Christopher Knight, Sally Mulholland

**Affiliations:** 1 Department of Nutrition and Dietetics, School of Medicine, Flinders University, Adelaide, Australia; 2 Human Nutrition Section, Division of Developmental Medicine, University of Glasgow, UK; 3 Robertson Centre for Biostatistics, University of Glasgow, UK; 4 Moredun Research Institute, Penicuik, UK; 5 Faculty of Life Sciences, University of Copenhagen, Denmark

## Abstract

**Background:**

Lactational mastitis is a painful, debilitating condition that if inappropriately managed, may lead women to discontinue breastfeeding prematurely. The aim of this paper is to report the incidence of mastitis in the first six months postpartum in a Scottish population, its impact on breastfeeding duration and to describe the type and appropriateness of the support and management received by affected women from health professionals.

**Methods:**

A longitudinal study of 420 breastfeeding women was undertaken in Glasgow in 2004/05. Participants were recruited and completed a baseline questionnaire before discharge from hospital. Cases of mastitis were reported either directly to the researchers or were detected during regular follow-up telephone interviews at weeks 3, 8, 18 and 26. Women experiencing mastitis provided further information of their symptoms and the management and advice they received from health professionals.

**Results:**

In total, 74 women (18%) experienced at least one episode of mastitis. More than one half of initial episodes (53%) occurred within the first four weeks postpartum. One in ten women (6/57) were inappropriately advised to either stop breastfeeding from the affected breast or to discontinue breastfeeding altogether.

**Conclusion:**

Approximately one in six women is likely to experience one or more episodes of mastitis whilst breastfeeding. A small but clinically important proportion of women continue to receive inappropriate management advice from health professionals which, if followed, could lead them to unnecessarily deprive their infants prematurely of the known nutritional and immunological benefits of breast milk.

## Background

Lactational mastitis is a painful, debilitating condition that can adversely affect mothers in their efforts to breastfeed their babies [1,2]. Despite being a relatively common complication of lactation, surprisingly few studies documenting the incidence of, and risk factors for, the condition have been reported. To our knowledge no large longitudinal study of mastitis has been conducted in the UK in recent times. Observational studies conducted in the USA [3], Finland [4] New Zealand [5] and Australia [6,7] suggest however, that up to 20–25% of breastfeeding women will develop mastitis during the course of lactation and approximately 20–35% of women who develop mastitis will experience recurrent episodes [5-8].

In some studies, mastitis has been associated with the premature cessation of breastfeeding [1,2]. Sufferers of mastitis may stop breastfeeding because of the pain associated with the condition or because they have been inappropriately advised by a health professional to do so. Health professionals therefore, have a responsibility to provide appropriate advice and support to assist women to manage the condition successfully and to continue breastfeeding.

The purpose of this paper is to report the incidence of mastitis in the first six months postpartum in a Scottish population, its impact on breastfeeding duration and to describe the type and appropriateness of the support and management received by affected women from health professionals.

## Methods

Study participants were recruited from women who had given birth at the Princess Royal Maternity Hospital in Glasgow between April 2004 and January 2005. Women who were breastfeeding at the time of recruitment and had delivered a full-term, healthy singleton were eligible to participate. Those who were fully formula feeding at the time of recruitment, lived outside the Greater Glasgow Conurbation, had a multiple delivery, a low birth weight infant (< 2500 g), a premature delivery (< 37 weeks gestation) or who could not read and speak English were excluded from the study.

Women meeting the inclusion criteria were visited on the maternity ward by the research midwife (SM) within 24–48 hours of delivery. They were given a written and verbal description of the study and were informed that they could decline to participate in, or withdraw from, the study without prejudice to their care. The study was approved by the Local Research Ethics Committee of the North Glasgow University Hospitals NHS Trust and written informed consent was obtained from participants.

Participants completed a baseline questionnaire prior to or shortly after discharge from hospital, providing information on demographic characteristics and previous births, breastfeeding experience and episodes of mastitis. Women were followed up by telephone interview at 3, 8, 18 and 26 weeks or up to the time that they discontinued breastfeeding. Follow-up interviews included questions on breastfeeding practices, breast and feeding related problems, breast care, use of breast pumps and maternal and infant health.

### Case definition

Mastitis was defined as a red, tender, hot, swollen area of the breast, accompanied by one or more of the following [6]:

i) an elevated temperature (either estimated or measured as being ≥ 38°C ) or

ii) one or more of the constitutional symptoms of fever (body aches, headaches and chills) or

iii) diagnosis of mastitis from a medical practitioner.

Symptoms had to have been present for a minimum duration of 24 hours [7].

Women who experienced an episode of mastitis post-discharge were instructed to telephone the research team to inform them of the event if their symptoms persisted for 24 hours or more. Additional cases of mastitis were identified retrospectively at the time of the telephone follow-up interviews. Those identified by either method as having mastitis were requested to complete and return a 'Mastitis Case' questionnaire, a copy of which had been given to them as part of an information pack at the time of recruitment (see Additional file). This questionnaire elicited information about events leading up to the episode of mastitis, management advice received and followed, and outcome.

### Statistical analysis

Data were analysed using the Statistical Package for the Social Sciences (SPSS for Windows Version 15) [9]. The estimated incidence of mastitis was based on women who had symptoms meeting our criteria who either directly contacted the research team or who were identified at one of the follow-up interviews. The target sample size of 500 had the power to detect an incidence of mastitis of 20% with an accuracy of 3.5%. The achieved analysis population of 420 had the power to detect an incidence of 20% with an accuracy of 3.8%.

Descriptive statistics include means and standard deviations or percentages. Survival analysis and the log-rank test were performed to determine if there was a difference in the duration of breastfeeding between women who developed mastitis and women who did not.

## Results

During the study period 1141 breastfeeding women were identified. Of these, 183 were missed or discharged prior to completion of recruitment and 372 did not meet the eligibility criteria. The remaining 586 women were invited to participate and of these 40 women refused to participate, a further 46 agreed to participate but provided incorrect or insufficient follow-up contact details and a further 80 discontinued breastfeeding within two weeks of delivery. In most cases women discontinuing breastfeeding before 2 weeks failed to establish breastfeeding successfully due to feeding difficulties. As an objective of the study was to identify determinants of mastitis (to be reported separately), any woman discontinuing breastfeeding before 2 weeks for reasons other than mastitis was excluded from the analysis population as their inclusion might reduce or mask the association of feeding difficulties and mastitis.

### Incidence of mastitis

**Figure 1:**
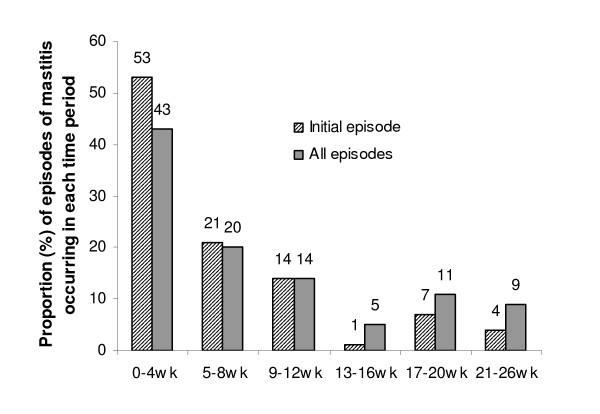
Proportion (%) of episodes of mastitis occurring in each time period (n = 57).

In total, 420 women were included in the analysis population (response rate 72%) and 74 women (18%, 95%CI: 14%, 21%) were identified as having experienced at least one episode of mastitis in the first 26 weeks postpartum. Mastitis was not significantly more common in women with a prior history of mastitis compared with those with no prior history (Table 1). Fifty-seven women (77% of cases) completed the Mastitis Case questionnaire, 39 of whom (68%) reported only one episode of mastitis but 13 women (23%) reported two episodes and five women (9%) three or more episodes of mastitis. In total 76 episodes of mastitis were reported by the 57 women completing the Mastitis Case Questionnaire. Thirty initial episodes (53%) of mastitis, and 33 (43%) of all episodes, occurred within the first 4 weeks postpartum (Figure 1). The mean time to first episode of mastitis was 6.3 (SD 6.6) weeks.

**Table 1:** Characteristics of women (n = 420)

**Characteristics**	**No mastitis (n = 346)**		**Mastitis (n = 70)**		**Chi-square test p value**
	**n**	**%**^1^	**N**	**%**^1^	
Age (years)					
<25	34	10	11	16	0.106
25–29	84	26	11	16	
30–34	118	36	22	31	
≥ 35	89	27	26	37	
Marital status					
Married or living with partner	306	94	66	96	0.623
Other	19	6	3	4	
Deprivation category^2^					
1–2	69	20	24	32	0.044
3–5	164	47	33	45	
6–7	113	33	17	23	
Education					
Did not complete high school	49	15	12	17	0.622
Completed high school	14	4	4	6	
Professional or technical Diploma/certificate	145	45	34	49	
University degree	115	36	19	27	
Number of children					0.405
1	184	57	33	48	
2	96	29	24	35	
≥ 3	45	45	12	17	
Previous history of mastitis					
First time mother	184	57	33	48	0.447
Parity > 1, never breastfed before	13	4	3	4	
Breastfed before, no mastitis	95	29	22	32	
Mastitis in past	33	10	11	16	

^1 ^Percentages may not total 100 due to rounding

^2 ^Where 1 = least deprived group and 7 = most deprived

### Mastitis and breastfeeding duration

**Figure 2:**
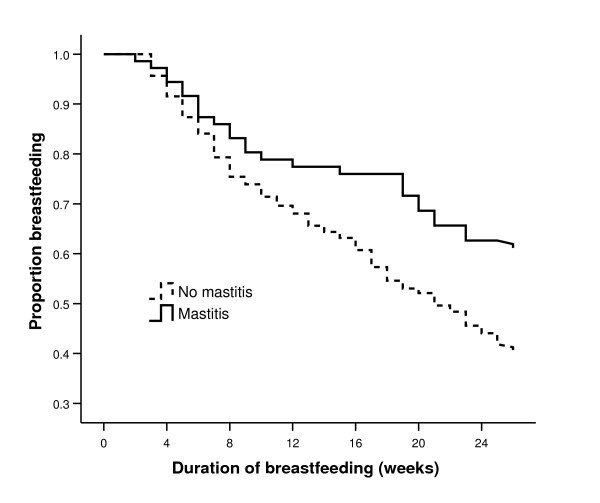
Duration of breastfeeding, by mastitis (n = 420).

Of the 74 women who developed mastitis 28 (38%) stopped breastfeeding before the end of the study (26 weeks). The duration from time of mastitis to stopping breastfeeding was 6.1 (SD 6.6) weeks. Of the other 46 women who developed mastitis, 41 were continuing to breastfeed at 26 weeks while the remaining 5 were lost to follow-up during the study but were breastfeeding at the time of last study contact. The occurrence of mastitis was not negatively associated with breastfeeding duration (Figure 2). In fact, those women who suffered an episode of mastitis were significantly more likely to be breastfeeding at 26 weeks than those who did not suffer mastitis (Log-rank test χ^2 ^= 8.81, df = 1, p = 0.003).

### Advice and treatment

Information on advice and treatment was available for the 57 women who completed the Mastitis Case questionnaire. Of these women, 21 (37%) were able to manage their first episode of mastitis without consulting a health professional. Thirty six women sought advice from a health professional: 21 (37%) from their General Practitioner (GP), 18 (32%) from a Community Midwife and 12 (21%) from a Health Visitor. Twelve women (21%) consulted more than one health professional, possibly indicating that more serious cases were referred on to a woman's GP by her Community Midwife or Health Visitor.

Thirty (53%) of those women who completed a questionnaire and 78% (28/36) of women who reported consulting a health professional were prescribed an antibiotic. Of these, nine women (30%) were prescribed flucloxacillin, five (17%) amoxicillin, two (7%) erythromycin, two (7%) co-amoxiclav (amoxicillin and clavulanic acid), one (3%) ceftriaxone (a third generation cephalosporin) and one (3%) co-fluampicil (ampicillin and flucloxacillin). Ten women (33%) could not recall what antibiotic they were prescribed.

The type of advice given to women by their health professional is listed in Table 2. Six women (10%) were told to stop feeding either from the affected breast or altogether.

**Table 2:** Advice received by women for management of mastitis from a health professional (n = 57)

**Advice**	**n**	**%**
Feed frequently from the affected breast	31	54
Feed frequently from the affected breast first/empty the affected breast	30	53
Express milk between feeds or when infant not interested in feeding	4	7
Do NOT stop breastfeeding	1	2
Stop breastfeeding or discontinue feeding from the affected breast	6	10

Percentages add to more than 100 as some women may have received more than one form of advice

## Discussion

The incidence of mastitis reported in this study is comparable to the incidence rates of 17.3% and 20% reported in recent Australian studies [1,6,10]. The only other study of Scottish women identified was conducted in the 1940s and reported an incidence of breast abscess of 8.6% [11]. However, as non-suppurative forms of mastitis were not identified, the real incidence of mastitis was likely to be higher. This and other earlier studies [8,12] probably underestimated the true incidence due to limitations in case ascertainment and the short time period that women were followed postpartum. For instance, earlier studies identified only those women with mastitis who sought medical treatment from the hospital where they were delivered and used hospital medical records as their source of data [8,12]. A more recent US study [3] reported a 9.5% incidence of health care provider-diagnosed lactational mastitis. In our study only one woman reported visiting a hospital casualty department with approximately one third of women visiting their GP. The majority of women either self-managed their mastitis or consulted only their community midwife and/or health visitor for management advice. In general, incidence rates for mastitis are below 10% when medical records and women seeking medical advice are used as a source of data, whereas incidence rates of around 20% are seen in studies where diagnosis is based on self-reported symptoms [1].

The recommended management of mastitis is usually conservative, the key recommendation being that mothers continue to breastfeed and to feed more frequently or express milk from the affected breast(s), in an effort to clear blocked ducts and engorgement [13-15]. Most women received advice consistent with current recommendations, however one in ten affected women were inappropriately advised by a health professional either to stop breastfeeding from the affected breast or to stop breastfeeding altogether. While in relative terms this may seem small, in absolute terms this represents approximately 680 women across Scotland receiving inappropriate advice annually. This estimation is based on the assumption that 70% of Scottish women delivering in 2004 [16] (n = 53957) initiated breastfeeding [17] and that 18% of these women experienced mastitis, of whom 10% received inappropriate advice.

There appear to be differences between countries in the extent to which antibiotics are prescribed to treat mastitis. In our study just over half (53%) of women who reported mastitis were prescribed antibiotics. This is higher than the 38% reported in a Finnish study [4] but lower than the 75% or more in Australian studies [6,18] and 86% in a recent US study [3]. In a recent Swedish study just under 15% of women with mastitis were prescribed antibiotics [19]. Of these, 3.3% of cases were prescribed antibiotics on the basis of their symptoms and the remaining cases (11.4%) were prescribed antibiotics on the basis of culture results.

The bacteriological analysis of breast milk is not routinely practiced in the UK with women usually being prescribed antibiotics on the basis of the severity and duration of their symptoms. Potentially pathogenic bacteria are found in the breast milk of healthy breastfeeding women and because the results from bacterial cultures may be difficult to interpret, it has been suggested that the bacteriological examination of breast milk is not particularly informative in the decision to treat mastitis with antibiotics [19]. However, in light of the fact that community acquired methicillin resistant *Staphylococcus aureus *(MRSA) is becoming more common, breast milk culture and sensitivity testing is recommended if the condition does not respond to antibiotic therapy within two days or if the mastitis recurs [14,15].

The difference in prescribing rates may be related to the number of women who self-manage their condition or seek advice from a heath professional other than their GP. Scottish women tended to consult their Community Midwife or Health Visitor, some of whom may have organised a prescription for antibiotics, with only just over a third consulting their GP. Whereas in an Australian study the majority of women (73%) had sought treatment and advice from their GP [6] and all of the women in a US study [3] were diagnosed following a medical consultation, thus increasing the likelihood of antibiotics being prescribed.

*Staphylococcus aureus *is the most common organism responsible for mastitis [20] and recent Clinical Practice Guidelines [15,21] recommend penicillinase-resistant penicillins such as flucloxacillin and dicloxacillin as the drug of first choice, or cephalexin and clindamycin in women who are allergic to penicillin. While the WHO publication on mastitis also recommends amoxicillin and erythromycin [14] more recent guidelines advise against the use of these drugs on the basis that a significant proportion of isolates of *Staphylococcus aureus *are resistant to these antibiotics [15,21]. Of the women who could recall the antibiotic they were prescribed (20/30) almost half (9/20) were prescribed an antibiotic that was not consistent with current practice guidelines. Kvist et al. recommend that the "imprudent use of antibiotics be avoided because of the spread of MRSA and other multi-resistant pathogens" [19]. Both their and our results suggest that a relatively large proportion of women can conservatively manage their mastitis without resorting to taking antibiotics.

Mastitis has been associated with the premature cessation of breastfeeding [1,2]. However, this was not the case in a recent study of Australian women where no association between mastitis and breastfeeding duration was found [10]. In our study, women who experienced mastitis were significantly more likely to be breastfeeding at 26 weeks than those who did not experience mastitis, which is similar to the finding of a study of New Zealand mothers [5]. Vogel et al. concluded that mastitis is more likely to occur in mothers with ample milk supply, who may be more at risk of milk stasis if they delay or miss a feed [5].

The strengths of this study are the relatively high response (72%) and the high follow-up rate (95%). In addition, we had frequent and regular contact with women allowing us to pinpoint the timing of onset of mastitis. There are also a number of limitations to this study. Firstly, women identified as cases through the follow-up interviews were only identified if they answered yes to having had mastitis specifically. They were not asked if they had experienced any symptoms suggestive of mastitis. However, the results of our study are strikingly similar to those of Amir et al. who, in order to reduce bias, avoided asking about mastitis directly but collected information about mastitis symptoms [10]. A further limitation of this study is that almost half of participants were continuing to breastfeed at 6 months, compared with the national average of 25% [17], suggesting that our sample was not necessarily representative of all breastfeeding women in Scotland. Despite these limitations, the mastitis incidence rate in this study is reasonably consistent with the incidence rates from studies of women in other Western countries.

## Conclusion

The findings of this study suggest that one in six women may develop lactational mastitis. While most women receive appropriate management advice from health professionals, a clinically significant number of women are advised to stop breastfeeding from the affected breast or to stop breastfeeding altogether. If followed, this advice could lead to women unnecessarily depriving their infants prematurely of the known nutritional and immunological benefits of breast milk.

## Competing interests

The authors declare that they have no competing interests.

## Authors' contributions

JAS designed the study, analysed data and drafted the manuscript. MR analysed data. JF and CK contributed to the design of the study. SM collected the data. All authors revised the manuscript and approved the final version.

## Supplementary Material

Additional file 1 Mastitis Case Questionnaire. Click here for file
