# Supplementary material for: Occurrence of lactational mastitis and medical management: A prospective cohort study in Glasgow
Source: Int Breastfeed J. 2008 Aug 25;3:21. doi: 10.1186/1746-4358-3-21 (PMC2542350; doi:10.1186/1746-4358-3-21)
Supplement: Additional file 1 — Mastitis Case Questionnaire. [file 1746-4358-3-21-S1.doc]

#
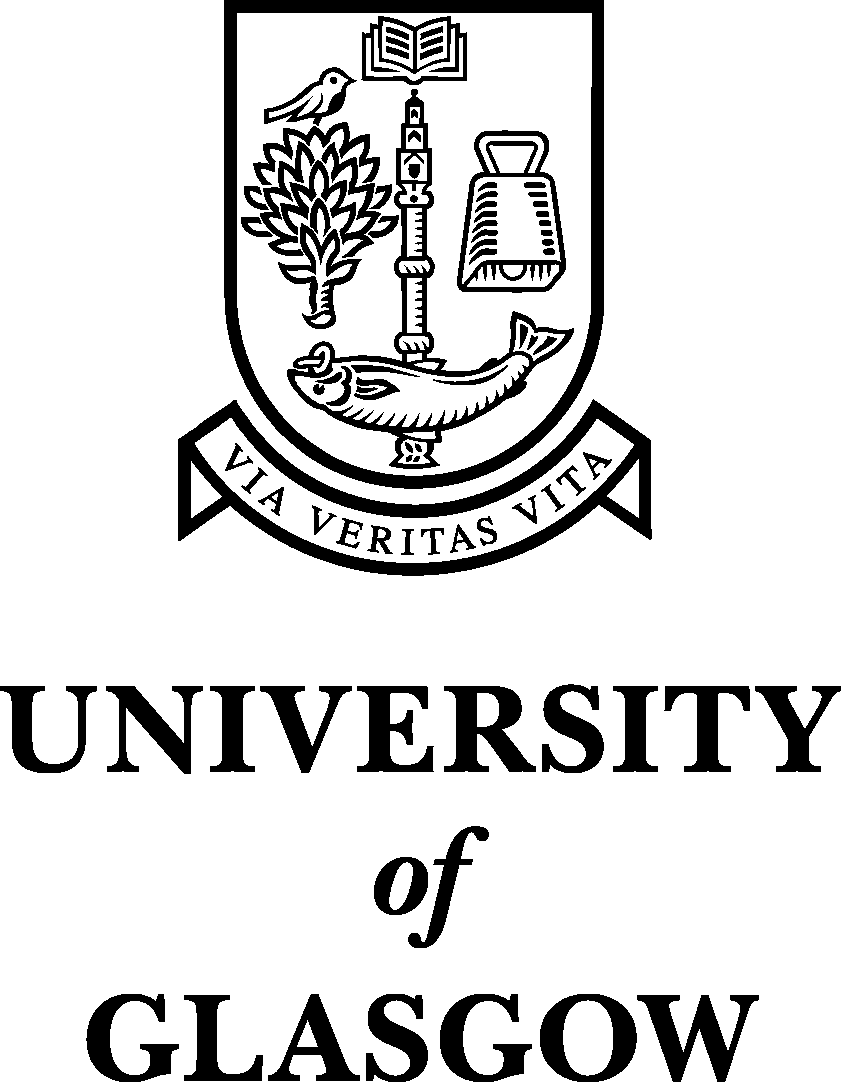


# MASTITIS STUDY

**MOTHER’S MASTITIS QUESTIONNAIRE**

This is the questionnaire that we would like you to fill in if, and when, you have an episode of mastitis. Please contact **Sally Mulholland** on **0141 211 5019** within 12 to 24 hours of you developing mastitis. She will discuss with you the arrangements for collecting a sample of your breast milk. This questionnaire should be completed once your mastitis has got better.

This questionnaire is designed to investigate the risk factors that might be associated with mastitis. It has been divided into three sections. Section 1 contains questions about the symptoms and treatment associated with your most recent bout of mastitis. Section 2 contains questions concerning your breastfeeding history. Section 3 contains questions about you and your baby.

Most of the questions can be answered by circling or ticking the answer that applies to you.

FOR EXAMPLE:

What colour is your hair?

Brown 1

Red 2

Blonde 3

Grey 4

I’m not sure 5

Which sport does your child enjoy playing?

YES NO

Football 1 2

Cricket 1 2

Tennis 1 2

Swimming 1 2

Some questions ask you to rate your answer on a scale of 1 to 5.

FOR EXAMPLE:

Please rate how much your child enjoys playing football.

1 2 3 4 5

hates playing loves playing

football football

Some questions require a written answer in the space provided.

This questionnaire will take approximately 15-20 minutes to complete. If you wish to write any further comments please do so at the bottom of the last page. **Please return the questionnaire to us in the pre-paid envelope provided.**

**SECTION 1**

**THIS SECTION IS ABOUT THE SYMPTOMS AND TREATMENT ASSOCIATED WITH YOUR MOST RECENT EPISODE OF MASTITIS.**

1. How many times have you had mastitis when breastfeeding THIS baby?

1

2

3

4

other (please indicate) ___________________________

2. About how old was your baby when you first experienced the symptoms associated with this bout of mastitis?

______________ weeks

PLEASE RATE HOW YOUR BREAST FELT AT THE HEIGHT OF YOUR MASTITIS ON A SCALE OF 1 TO 5.

3. The area of my breast affected by the mastitis was:

1 2 3 4 5

normal unbearable

tenderness to touch

1 2 3 4 5

normal skin skin was very

temperature hot to touch

1 2 3 4 5

normal very red

appearance and swollen

4. Please shade the area on the diagram to show which area of your breast(s) were affected by your mastitis

# Right breast Left breast

Left upper

quadrant (near

left arm pit)

Right upper

quadrant (near

right arm pit)

5. How long did it take before your breasts returned to normal? __________ days

6. At the height of your mastitis was your temperature:

Below 37.4^o^C 37.5 to 38 ^o^C 38.1 to 38.5 ^o^C 38.6 to 40 ^o^C over 40 ^o^C

IF YOU DON’T KNOW what your temperature was please estimate using the following scale

Normal slightly elevated high very high extremely high

Below 37.4^o^C 37.5 to 38 ^o^C 38.1 to 38.5 ^o^C 38.6 to 40 ^o^C over 40 ^o^C

7. If your temperature was elevated, how long did it take for your temperature to return to normal?

Within 24 hours 24-48 hours more than 48 hours

8. Did you experience chills associated with your mastitis?

1 2 3 4 5

Not at all Severe shaking

and chills

9. Did you experience flu like aching?

1 2 3 4 5

Not at all Severe flu

Like aching

10. Did you feel so ill you were confined to bed? YES 1

NO 2

11. If YES, please circle the length of time:

Less than 24 hours 24-48 hours more than 48 hours

12. Did you receive advice regarding the treatment of your mastitis from:

YES NO

General Practitioner 1 2

Midwife 1 2

Health visitor 1 2

La Leche League 1 2

Other (please specify) ______________________________

13. If you received advice from more than one source, was the information conflicting:

1 2 3 4 5

not at all extremely

conflicting conflicting

14. Please tick whether the following information was given in the treatment of your mastitis. If YES, please state the source of this information.

|  | | YES | NO | SOURCES  (e.g. GP, health visitor, midwife, La Leche Counsellor, your mother) |
| --- | --- | --- | --- | --- |
| Stop feeding from the affected breast | | 1 | 2 |  |
| Wean your baby | | 1 | 2 |  |
| Feed frequently from the affected breast | | 1 | 2 |  |
| Feed from the affected breast first | | 1 | 2 |  |
| Massage the affected area prior to and during a feed | | 1 | 2 |  |
| Discontinue the use of nipple lotions or creams | 1  Not  Applicable | | 2  3 |  |
| Apply heat to the affected area prior to and during a feed | | 1 | 2 |  |
| Feed with the baby’s chin towards the affected area | | 1 | 2 |  |
| Apply cold packs after feeding | | 1 | 2 |  |
| Have ultrasound treatment to the affected area | | 1 | 2 |  |

Other (please describe)

15. Please tick which of the following steps you took to resolve your mastitis. IF YES, please show how effective you felt this treatment was in helping treat the problem.

**YES NO** **EFFECTIVENESS OF TREATMENT**

Did you:

Stop feeding from 1 2 1 2 3 4 5

the affected breast not at all very effective

Wean your baby 1 2 1 2 3 4 5

not at all very effective

Feed frequently 1 2 1 2 3 4 5

from the affected breast not at all very effective

Feed from the 1 2 1 2 3 4 5

affected breast first not at all very effective

Massage the affected 1 2 1 2 3 4 5

area prior to and not at all very effective

during a feed

Apply heat to the 1 2 1 2 3 4 5

affected area prior to not at all very effective

and during a feed

Feed with the baby’s 1 2 1 2 3 4 5

chin toward the not at all very effective

affected area

Apply cold packs 1 2 1 2 3 4 5

after feeding not at all very effective

Have ultrasound 1 2 1 2 3 4 5

treatment to the not at all very effective

affected area

Other (please describe)

16. Were you given intravenous antibiotics to treat your mastitis? YES 1

NO 2

17. Were you given antibiotic (intramuscular) injections to treat your YES 1

mastitis? NO 2

18. Were you prescribed antibiotic tablets to treat your mastitis? YES 1

NO 2

IF YES, what was the name(s) and doses(s) of the antibiotic tablet prescribed?

______________ _______ ___________________ _________________

name of antibiotic dose how many times per day for how many days?

______________ _______ ___________________ _________________

name of antibiotic dose how many times per day for how many days?

19. Did you finish the prescribed course of antibiotics? YES 1

NO 2

20. Did the person(s) you sought advice or treatment from attempt to YES 1

determine the cause of the mastitis? NO 2

21.If YES, what was the reason given for your mastitis?

22. What do you think was the cause of your mastitis?

**SECTION 2**

**THIS SECTION HAS QUESTIONS CONCERNING YOUR BREASTFEEDING HISTORY**

23. IN THE WEEK BEFORE YOU DEVELOPED MASTITIS:

| Did you experience trauma to your breast from strenuous exercise? | 1  No injury | 2 | 3 | 4 | 5  severely  traumatised |
| --- | --- | --- | --- | --- | --- |
| Did you experience trauma to your breast from injury? (e.g. kick from toddler, hand expressing, rough foreplay)? | 1  No injury | 2 | 3 | 4 | 5  severely  traumatised |
| Did you experience trauma to your breast from cracked or grazed nipples? | 1  No injury | 2 | 3 | 4 | 5  severely  traumatised |
| Did you use a nipple shield when feeding? | 1  Never | 2 | 3 | 4 | 5  Always |
| Did you use nipple airers in between feeds? | 1  Never | 2 | 3 | 4 | 5  Always |
| Did you use breast pads after feeds? | 1  Never | 2 | 3 | 4 | 5  Always |
| Did you apply nipple creams or lotions? | 1  Never | 2 | 3 | 4 | 5  Always |
| Did you generally feel more stressed than normal? | 1  not more stressed | 2 | 3 | 4 | 5  extremely more stressed |
| Did you generally feel more tired or run down, than normal? | 1  not more tired | 2 | 3 | 4 | 5  extremely tired |

24. What is your baby’s preferred side for feeding?

left breast 1

right breast 2

no preference 3

25. IN THE 48 HOURS BEFORE YOUR MASTITIS:

Did you suffer from:

| Engorgement? | 1  Not at all | 2 | 3 | 4 | 5  extremely engorged |
| --- | --- | --- | --- | --- | --- |
| Did you suffer from blocked ducts? | 1  Not at all | 2 | 3 | 4 | 5  extreme blockage |
| Did your milk appear: | 1  the same as usual | 2 | 3 | 4 | 5  thicker than usual |
| Did you feed your baby? | 1  much less than usual | 2 | 3  same as usual | 4 | 5  much more than usual |
| Did you give your baby complementary formula? | 1  never | 2 | 3  same as usual | 4 | 5  much more than usual |
| Did you breastfeed your baby  according to a preset routine? | 1  never | 2 | 3 | 4 | 5  always |
| Did you have to delay your baby’s breastfeeds? | 1  never | 2 | 3 | 4 | 5  always |
|  |  |  |  |  |  |
| Did you experience restriction to any part of your breasts from: |  |  |  |  |  |
| a tight bra? | 1  no restriction | 2 | 3 | 4 | 5  severely restricted |
| tight clothing? | 1  no restriction | 2 | 3 | 4 | 5  severely restricted |
| Did you wear a bra to sleep at night? | 1  never | 2 | 3 | 4 | 5  always |
| Was your baby difficult to attach to the breast? | 1  never | 2 | 3 | 4 | 5  always |
| Did your nipple generally hurt during a feed? | 1  never | 2 | 3 | 4 | 5  always |
| Did you have to depress your breast with your finger to allow your baby room to breathe? | 1  never | 2 | 3 | 4 | 5  always |
| Immediately after a breastfeed was your nipple generally: | 1  normal shape | 2 | 3 | 4 | 5  extremely misshapen |

# SECTION 3 SOME QUESTIONS ABOUT YOUR BABY AND YOU

26. Has your baby been diagnosed with any of the following conditions since birth?

tongue tie 1

high palate 2

sucking disorder 3

none of the above 4

**IN THE WEEK BEFORE YOUR MASTITIS:**

27. Did your baby suffer from:

thrush of the mouth 1

thrush of the anal or genital area 2

neither of these 3

28. Did you baby suffer from any illness? YES 1

NO 2

29. If YES, please describe

30. Do you have anaemia? YES 1

NO 2

Not that I am aware of 3

31. Were you sick in the week before your mastitis?

(e.g. flu. Cold, asthma etc.?) YES 1

NO 2

If YES, please describe

32. Did you suffer with any thrush infection in the week before your mastitis?

YES 1

NO 2

1. Were you taking any medications at the time of the onset of your mastitis? (please include oral contraceptives, medicines prescribed by your doctor, over the counter medicines and any medicines or supplements from a health food store).

**THANK YOU FOR COMPLETING THIS QUESTIONNAIRE**

**PLEASE RETURN IT IN THE PREPAID ENVELOPE PROVIDED**
